# Hepatoprotective Activity of the Ethanolic Extract of *Ficus carica*Linn. LeavesinCarbon Tetrachloride-Induced Hepatotoxicityin Rats

**Published:** 2011

**Authors:** Mohd Mujeeb, Shah Alam Khan, Vidhu Aeri, Babar Ali

**Affiliations:** a *Department of Pharmacognosy and Phytochemistry, Faculty of Pharmacy, Jamia Hamdard, New Delhi-110062, India.*; b * Department of HIT, King Abdulaziz University, Jeddah, Kingdom of Saudi Arabia.*

**Keywords:** *Ficus carica*, Hepatoprotective activity, Carbontetra chloride, Silymarin

## Abstract

The ethanolic extract of *Ficus carica *leaves was screened for hepatoprotective and antioxidant activity in hepatotoxic Albino rats induced via carbon tetrachloride. The degree of protection was measured by estimating biochemical parameters such as serum glutamate Oxaloacetate transaminase (SGOT), serum glutamate pyruvate transaminase ( SGPT),totalprotein (TP), totalalbumin (TA), alkaline phosphatase (ALKP) and the level of total serum bilirubin. The extract in addition reduced CCl_4_ induced lipid peroxidation i*n-vivo *and *in-vitro*. The ethanolic extract (50 mg/kg, 100mg/kg,200mg/kg)exhibited significant hepatoprotection incarbontetra chloride in toxicated rats in a dose dependant manner. The hepatoprotective effects of the extract were comparable with the standard drug silymarin 10)mg/kg body weight, IP).

## Introduction

The Liver is a key organ in the human body, regulating homeostasis and is a frequent target for a number of toxicants (1). In spite of tremendous scientific advancement in the field of hepatology during recent years, liver problems are on the rise. Regrettably there are only a few drugs with serious side effects available for the treatment of liver ailments (2). In view of the undesirable side effects of synthetic agents, there is growing focus towards the therapeutic evaluation of medicinal plants using systemic research methodology. 

*Ficus carica* Linn. (Family; Moraceae) commonly known as Fig is a small or moderatelysized deciduous tree indigenous to asia minor, persia, syria and the mediterranean region (3). The plant leaves has been reported to contain furanocoumarins such as psoralen, bergapten, xanthotoxin (4), triterpenes such as calotropenyl acetate, lupeol acetate (5), isoschaftoside (6) and certain sterols.

Fig leaves have been traditionally used in the treatment of vitiligo, diabetes, coughs, asthma, constipation and gingivitis (3, 7). The other reported pharmacological activities of fig leaves include cytotoxic (8), hypoglycemic (9) and anthelmintic activity (10).

With a view that sterols, flavonoids, furanocoumrains and triterpens found in medicinal plants are used as hepatoprotective drugs (11), it was thought worthwhile to conduct hepatoprotective studies on the leaves of the *Ficus carica* plant in a scientific manner to validate its use in the traditional system of medicine.

## Experimental

Plant material

Leaves of the *Ficus carica* were collected from Jamia Hamdard (Hamdard university) campus, New Delhi, India and authenticated by the taxonomist at the Department of Botany, Faculty of Science, Hamdard University. A voucher specimen was deposited in the herbarium of University for future reference.

Preparation of extract

Air dried and coarsely powdered leaves of the plant (1 kg) were soxhlet extracted with ethanol for 72 h. The ethanolic extract was then concentrated on a water bath and dried under reduced pressure to achieve a dark brown mass (95 g; yield- 9.5%).

Preliminary phytochemical screening 

On preliminary phytochemical screening using the reported method (12), the ethanolic extract of the *Ficus carica *leaves showed positive tests for glycosides, steroids, triterpens and flavonoids.

*Determination of phenolic compounds*

The total phenols were estimated by the Folin-Ciocalteu reagent according with the method of Gao *et al.* (13). The total content was expressed as mg of gallic acid equivalents/g extract.

*Determinaton of total flavonoids*

The total flavonoid content was estimated according to Kosalec *et al*. (14). The total content was expressed as mg of quercetin equivalents/g extract.

*Animals*

Hepatoprotective activity was carried out on Albino rats of either sex (110-145 g), supplied by the central animal house facility of Jamia Hamdard, New Delhi (Registration no. 173/CPCSEA). The rats were maintained in a 12 h light/dark cycle at 25 ± 2°C. They were allowed free access to a standard pellet diet (Amrut Laboratory Rat Feed, Navamaharashtra, Pune, India) and water *ad libitum*. The study was approved by the ethics committee CPCSEA and ethical norms were strictly followed during all experimental procedures.

*Drugs and dosing schedule*

The animals were divided into six groups; group I (control), group II (CCl_4_ treated), group III (CCl_4_ + silymarin treated), group IV, V and VI (CCl_4_ + extract). Animals of groups II, III, IV, V and VI were administered 50% (v/v) CCl_4_ in olive oil in a single dose of 2 mL/kg body weight per day for 4 days via the S.C. route. Simultaneously but at different hours of the day, animals of groups III, IV, V and VI were fed with silymarin suspension (10 mg/kg body weight, IP) in addition to ethanolic extract in doses of 50 mg/kg, 100 mg/kg and 200 mg/kg body weight, IP for 4 days respectively. Animals of group I were given distilled water in a volume of 10 mL/kg body weight.

*Serum analysis*

On the day 5, after the treatment period all of the subject animals were anaesthetized and sacrificed. Blood was withdrawn from the heart and their serum was separated by centrifugation at 3000 rpm at 30^o^C for 15 min. This was subsequently analysed for various biochemical parameters including serum transaminases viz. SGOT (15), SGPT (15), total protein (16), total albumin, alkaline phosphatase (17) and total bilirubin content (18). 

*In-vitro antioxidant activity by DPPH method*

The stable 1, 1-diphenyl-2-picryl hydrazyl radical (DPPH) (75 μM) was used for the determination of free radical-scavenging activity of the extract (19). 2.95 mg of DPPH was dissolved in 100 mL of methanol to achieve a 75 μM DPPH solution.

**Table 1:** Effects of the ethanolic extract of Ficus carica leaves on biochemical parameters in CCl_4_ induced hepatotoxicity in rats

**Treatment**	**Dose** **(mg/Kg)**	**SGOT** **(U/mL)**	**SGPT** **(U/mL)**	**TB ** **(g/dL)**	**ALKP** **(U/mL)**	**TP** **(g/dL)**	**TA** **(g/dL)**
Control	-	133.80 ± 0.15	74.47 ± 0.04	0.65 ± 0.002	35.93 ± 0.09	14.17 ± 0.02	4.81 ± 0.01
CCl_4_	2 mL/Kg	177.76 ± 0.17	112.38 ± 0.05	2.29 ± 0.02	73.74 ± 0.01	7.81 ± 0.05	4.10 ± 0.02
Silymarin	10	133.88 ± 0.02^**^	85.30 ± 0.005^**^	0.65 ± 0.002^*^	37.18 ± 0.08^**^	10.65 ± 0.06^**^	4.73 ± 0.03^*^
Ficus carica	50	158.65 ± 4.25	104.25 ± 0.03^*^	1.17 ± 0.017	61.0 ± 0.028^*^	6.07 ± 0.012^*^	4.43 ± 0.01^*^
	100	151.00 ± 0.08^*^	98.84±0.05^*^	0.92 ± 0.002^*^	47.02 ± 0.05^*^	6.32 ± 0.06^**^	4.60 ± 0.04^*^
	200	141.45 ± 0.05^*^	90.71 ± 0.005^**^	0.74 ± 0.019^*^	35.12 ± 0.011^*^	7.21 ± 0.08^**^	4.65 ± 0.03^*^

Values are ± mean SEM. (n = 6)

^*^p < 0.05; ^**^p < 0.01 Vs CCl_4_. One-way analysis followed by Dunnet’s t-test.

SGOT: Serum glutamate oxaloacetate transaminase, SGPT: Serum glutamate pyruvate transaminase, TB: Total bilirubin,, ALKP: Alkaline phosphatise, TP: Total protein and TA: Total albumin.

**The ethanolic solution (0.5 mL) of plant extract in various concentrations was mixed in a test tube with 2.5 mL of methanol containing 75 μM DPPH giving a distinctive absorbance at 517 nm. The reaction mixture was set-aside in the dark at room temperature for 90 min and then its absorbance was recorded at 517 nm. Corresponding blank readings were also taken and the remaining DPPH was calculated. The experiment was repeated three times. Ascorbic acid and quercetin were used as standard controls.**

*In-vivo antioxidant activity*

The *in-vivo* antioxidant activity of the ethanolic extract was carried out in CCl_4_ intoxicated rats. The liver samples collected were washed with chilled normal saline, weighed and 10% (w/v) liver homogenate was generated in ice cold 0.15 M KCl solution using a motor driven Teflon pestle. The suspension was centrifuged at 2000 rpm at 4°C for 10 min and the clear supernatant was used for the estimation of the following antioxidant markers.

*Estimation of liver TBARS*

The measurement of Thiobarbituric acid reactive substances (TBARS) was carried out as an index of lipid peroxidation and measured in terms of malondialdehyde (MDA) content by following the method outlined by Ohkawa *et al. *(20). The total protein content in the tissue homogenate was determined using the method set out by Lowry *et al.* (21). The values of TBARS were presented as nmol MDA /mg protein. 

*Estimation of reduced glutathione (GSH)*

Glutathione was estimated using the Ellman reagent (5, 5-dithiobis-2-nitrobenzoic acid) according with Ellman’s method (22) and the attained values were expressed as μmoL/g of liver tissue.

*Statistical analysis*

The results of biochemical parameters are reported as mean ± SEM. The statistical significance was determined by means of a one-way analysis of variance (ANOVA) followed by Dunnet’s t-test (23). A p-value of < 0.05 was considered as being statistically significant.

## Results

HPTLC fingerprints of the ethanolic extract of *Ficus carica* leaves showed the presence of 20 spots, confirming the presence of a different class of phytoconstituents as revealed by the preliminary phytochemical screening. The total phenolic and flavonoid content was also determined. The overall phenolic and flavonoid content was found to be 135.8 ± 7.21 mg in gallic acid equivalent/g extract and 60.5 ± 5.2 mg inquercetin equivalent/g extract, respectively.

The administration of CCl4 led to significant hepatocellular damage as evident from the increase in serum activities of SGOT, SGPT, Alkaline phosphatase (177.76, 112.38, 73.74 units/mL, respectively) and total bilirubin (2.29 mg/dL). In addition to a decrease in the level of total protein (TP) and total albumin (TA) concentration (7.81, 4.10 g/dL, respectively) in comparison with the normal control group. Treatment of rats with the ethanolic extract of leaves at a dose of 50 mg/kg, 100 mg/kg and 200 mg/kg body weight i.p. exhibited a significant reduction (p < 0.01 and 0.05) in CCl4 induced elevation of serum GOT, GPT, ALKP (158.65–141.45, 104.25-90.71, 61-35.12 units/mL, respectively) bilirubin (1.17-0.94 mg/dL) and increased the level of TP and TA (6.07-7.21, 4.43- 4.65 g/dL, respectively) as illustrated by Table 1. Treatment with silymarin also significantly reversed the hepatotoxicity. However, the extract produced hepatoprotective activity in a dose dependant manner.

The IC_50_ of the ethanolic extract was found to be 72.9 µg/mL. The value was found to be comparable to that of standard ascorbic acid and quercetin. In the present study, administration of ethanolic extract at doses of 50, 100 and 200 mg/kg body weight to CCl_4_ intoxicated rats caused a significant increase in the level of GSH (35.3- 59.46 µmoL/g) and a significant decrease in the level of TBARS (1.47-0.615 nmol MDA/mg protein) in comparison with the CCl_4_ treated control group (Table 2). In the liver tissues standard Silymarin at a dose of 10 mg/Kg also showed significant antioxidant activity. The results elicited by the ethanolic extract were comparable with that of the standard drug silymarin.

**Table. 2:** Effects of the ethanolic extract of *Ficus carica* leaves on TBARS and GSH levels in the liver tissues of CCl_4_ intoxicated rats

**Treatment**	**Dose** **(mg/Kg)**	**TBARS** **(nmoL MDA/mg protein)**	**GSH** **(µmoL/g liver tissue)**
Control	-	0.65 ± 0.049	65 ± 12.22
CCl_4_	2 mL/Kg	1.95 ± 0.099^*^	13.12 ± 5.53^ *^
Silymarin	10	0.56 ± 0.08^c^	61.25 ± 11.23^ ***^
Ficus carica	50	1.47 ± 0.41^**^	35.3 ± 5.78^ **^
	100	1.33 ± 0.35^**^	40.38 ± 14.43^ **^
	200	0.615 ± 0.06^***^	59.46 ± 15.58^ ***^

Values are mean ± SEM (n = 6), ^*^ p < 0.001Vs Control

^**^p < 0.05; ^***^p < 0.001 Vs CCl_4_. One-way analysis followed by Dunnet’s t-test.

TBARS: Thiobarbutric acid reactive substances, GSH: Reduced glutathione*.*

## Discussion

CCl_4_ is one of the most commonly used hepatotoxins in experimental studies of liver diseases (24). The hepatotoxic effects of CCl_4_ are largely due to its active metabolite, trichloro methyl radical (25). These activated radicals bind covalently to macromolecules and induce peroxidative degradation of endoplasmic reticulum membrane lipids rich in polyunsaturated fatty acids. This leads to the formation of lipid peroxides, which in turn creates products such as malondialdehyde (MDA) that cause damage to the membrane. Lipid peroxidative degradation of the bio membrane is one of the principle causes of CCl_4_ toxicity, this is evidenced by the elevation of TBARS and a decrease in the activity of the free radical scavenging enzyme (GSH). In addition an elevation in the serum marker enzymes is also witnessed. The increase in the levels of serum bilirubin reflected the level of jaundice. An increase in transaminases was a clear indication of cellular leakage and a loss of the functional integrity of the cell membrane (26). The ethanolic extract significantly reduced the liver enzyme levels and increased the level of total serum protein in a dose dependant manner, indicating hepatoprotection. 

The DPPH test is a very convenient method for screening small antioxidant molecules as the intensity of reaction can be analysed by a simple spectrophotometric method (27). The DPH radical is scavenged by antioxidants through the donation of hydrogen to form stable radical DPPH molecules. The antioxidant radicals formed are stabilized through the formation of a non-radical product.

IC_50 _is considered to be a good measure of the antioxidant efficiency of pure compounds and extracts. The IC_50_ of the ethanolic extract of *Ficus carica* leaves (72.9 µg/mL) was found to be comparable to that of standard ascorbic acid and quercetin. 

Lipid peroxidative degradation of the biomembrane is one of the principle causes of CCl_4_ toxicity. This is evidenced by the elevation of TBARS and a decrease in the activity of free radical scavenging enzymes such as GSH in CCl_4_ intoxicated animals. Glutathione is a naturally occurring tripeptide and a non-enzymatic biological antioxidant that is abundant in many living creatures. It is widely known that a deficiency in GSH within living organisms can lead to tissue injury and disorder. The increase in the level of MDA in liver induced by CCl_4_ suggests enhanced lipid peroxidation. This leads to tissue damage and the failure of antioxidant defence mechanisms preventing the formation of excessive free radicals. Treatment with the ethanolic extract (50, 100 and 200mg/Kg) reduced the elevated level of TBARS (Thobarbituric acid reactive substances) in CCl_4_ intoxicated liver tissues. It was also observed that GSH depletion due to the CCl_4_ challenge was reversed by the test extract. 

Preliminary phytochemical screening indicated the presence of phenolic and flavonoid glycosides. The free radical mediated process has been implicated in the pathogenesis of different diseases. Thus the antioxidant activity of the ethanolic extract may be due to the presence of flavonoid and phenolic compounds. Its hepatoprotective activity is attributed to its antioxidant properties. Further isolation of the active principles responsible for its hepatoprotective activity is currently in progress within our laboratory.
